# Feasibility and acceptability of inserts promoting virtual overdose monitoring services (VOMS) in naloxone kits: a qualitative study

**DOI:** 10.1186/s12954-023-00792-z

**Published:** 2023-05-08

**Authors:** Fahad Safi, William Rioux, Nathan Rider, Barbara Fornssler, Stephanie Jones, S. Monty Ghosh

**Affiliations:** 1 Department of Medicine, Faculty of Medicine and Dentistry, University of Alberta, Edmonton, AB Canada; 2 Department of Medicine, Cumming School of Medicine, University of Calgary, Calgary, AB Canada; 3 School of Public Health, University of Saskatchewan, Saskatoon, Canada; 4 Three Hive Consulting, Vancouver, BC Canada; 5 Department of Internal Medicine, Faculty of Medicine and Dentistry, University of Alberta, Edmonton, AB Canada

**Keywords:** Opioid, Overdose, Naloxone, Public health, Virtual overdose monitoring services, Harm reduction, Naloxone kit inserts

## Abstract

**Background:**

In response to the ongoing opioid epidemic, there have been efforts to develop novel harm reduction strategies alongside scaling of currently implemented programs. Virtual overdose monitoring services (VOMS) are a novel intervention which aims to reduce substance-related mortality through technology for those who are out of reach of current supervised consumption sites. Scaling of naloxone programs presents a unique opportunity to promote VOMS to people at risk of substance-related mortality. This study aims to explore the feasibility and acceptability of naloxone kit inserts in promoting awareness of VOMS.

**Method:**

We used purposive and snowball sampling to recruit 52 key informants, including people who use drugs (PWUD) with experience using VOMS (*n* = 16), PWUD with no prior experience using VOMS (*n* = 9), family members of PWUD (*n* = 5), healthcare and emergency services professionals (*n* = 10), community-based harm reduction organizations (*n* = 6), and VOMS administrators/peer support workers (*n* = 6). Two evaluators completed semi-structured interviews. Interview transcripts were analyzed using thematic analysis informed to identify key themes.

**Results:**

Four key interrelated themes emerged, including the acceptability of naloxone kit inserts to promote VOMS, best practices for implementation, key messaging to include within promotional materials and facilitators to dissemination of harm reduction material. Participants highlighted that messaging should be promoted both inside and outside the kits, should be concise, provide basic information about VOMS and can be facilitated through current distribution streams. Messaging could further be used to draw attention to local harm reduction services and could be promoted on other supplies, including lighters and safer consumption supplies.

**Conclusion:**

Findings demonstrate that it is acceptable to promote VOMS within naloxone kits and highlight interviewees preferred ways to do so. Key themes that emerged from interviewees can be used to inform the dissemination of harm reduction information, including VOMS and bolster current strategies for reducing illicit drug overdose.

**Supplementary Information:**

The online version contains supplementary material available at 10.1186/s12954-023-00792-z.

## Introduction

In 2020, opioids accounted for 77% of drug-related deaths worldwide, with a record-high number of unintentional overdoses related to opioids being reported in Canada [1]. Over the course of the pandemic, Canada has seen a 96% increase in overdose deaths, a number which continues to rise [2]. This problem requires innovative solutions to prevent drug overdose among people who use drugs (PWUD). As an adjunct to traditional supervised consumption sites, online interventions have the potential to reduce drug overdose deaths and promote access to treatment, assessment, and prevention services [3, 4]. Scale-up of online interventions has been recommended in response to the unique challenges faced by PWUD as these services reduce stigma, remove geographic barriers, and decrease associated costs [5, 6].

One recently proposed solution aimed at reducing the harm associated with the overdose crisis is virtual overdose monitoring services (VOMS). These services can take the form of smartphone applications or telephone hotlines where an individual who is using substances can be monitored and emergency services or an emergency care plan can be activated if the person using substances becomes unresponsive [7]. Some VOMS available in Canada include the National Overdose Response Service, the BRAVE app, Connect by Lifeguard, and the Digital Overdose Response System app [7]. The National Overdose Response Service is a telephone hotline that connects an operator who will stay on the phone with the person using substances (the caller). The operator will check in with the caller continuously or periodically based on the caller’s preference and if the caller does not respond, the co-created safety plan is initiated, which may include engaging a close contact to support the client or utilizing emergency services (8). The Digital Overdose Response System is an app where PWUD register with their phone number and provide their location, then while using it alone, start a timer on the app and if the timer expires or the person needs help, the app initiates emergency services [9]. Additional technologies have been summarized in a recently published scoping review by Lombardi et al. [10].

Preliminary data evaluating the National Overdose Response Service indicate that VOMS can facilitate timely and anonymous access to emergency care for PWUD and that during the first 14 months of operations, the service monitored 2172 substance use events with 53 adverse events requiring emergency response and with no fatalities reported [7]. One study demonstrated that 68% of individuals with a cellphone would use an application to mitigate opioid-related drug overdose deaths [11]. Due to the relative infancy of these services, there is relatively little published data on their effectiveness; however, uptake is currently limited to urban areas which may already have access to supervised consumption services [7].

The widespread availability of naloxone kits [12, 13] reflects a unique opportunity to increase awareness of VOMS across Canada via naloxone kits and thereby increasing their efficacy. The distribution of naloxone kits is one of the key measures implemented across Canada to address the opioid crisis and prevent rising mortality [12, 13]. Every region in Canada offers free injectable naloxone which may be obtained through community pharmacies, shelters, medical centers, treatment centers, and emergency services such as police, paramedics, and/or firefighters [14]. Across the country, 8,700 naloxone distribution sites have distributed more than 590,000 naloxone kits, with more than 61,000 having been used to reverse an overdose [14]. Considering the widespread distribution of naloxone kits, providing information about VOMS in the form of inserts within naloxone kits might be an effective way to reach populations that use opioids and increase the general awareness of these services.

Rural communities experience a 45% higher rate of opioid-related overdose deaths than urban areas as well as disparity in naloxone administration by emergency medical services [15, 16]. Naloxone kits may also prevent overdose in rural communities which do not have access to other services [17]. The Government of Canada allocated $7,935,489 in March 2021 to the Canadian Red Cross in partnership with St. John Ambulance to expand naloxone access to rural, remote, isolated and otherwise underserved communities [18]. With the expansion of naloxone kits to remote locations, there is potential to reach various at-risk populations and provide them with access to harm-reduction services that are more accessible and will ultimately lead to an overall reduction in mortality due to opioid-related drug overdoses.

**Fig. 1:**
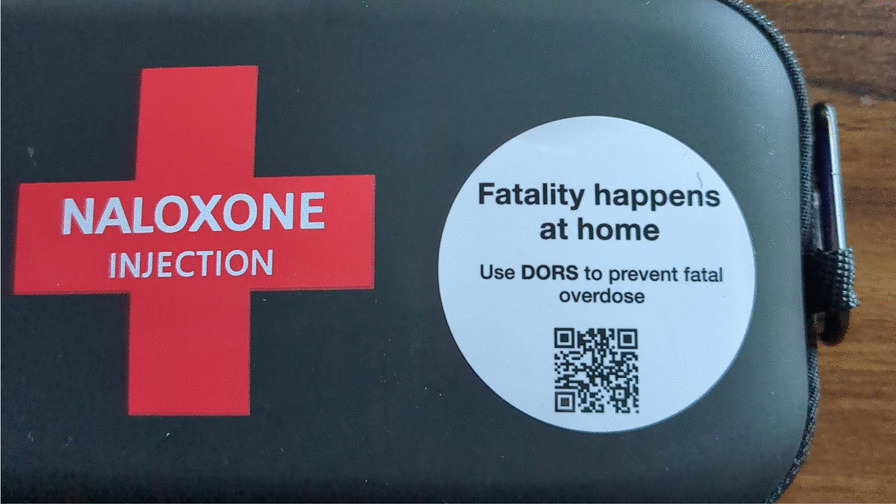
Naloxone kit messaging

In efforts to spread information about VOMS, a preliminary rapid literature review was conducted to determine any strategies that may be used to promote this novel service, however, provided no results. The use of messaging in naloxone kits was suggested as a potential avenue for the promotion of this service, as it has previously been used to disseminate other important harm-reduction material [19]. This strategy was rapidly implemented across the province of Alberta (Fig. 1), however, lacked community involvement and codesign, which are integral for addressing complex public health challenges [19]. In this study, we aim to understand the perspectives of multiple stakeholders, including PWUD, family members, health care professionals/emergency services, harm reduction organizations, and VOMS administrators, on how to best implement naloxone kit messaging.

## Methods

We conducted semi-structured in-depth interviews with 52 participants consisting of PWUD that have used VOMS previously (*n* = 16), PWUD who have not used VOMS previously (*n* = 9), family members of PWUD (*n* = 5), healthcare and emergency service professionals (*n* = 10), community-based harm reduction organizations (*n* = 6), and VOMS administrators (*n* = 6) all of which were relevant stakeholders in the distribution and use of naloxone kits. Eligible individuals were identified by using purposeful, snowball, and convenience sampling through existing peer networks and VOMS active in Canada (The National Overdose Response Service, Substance User Network of the Atlantic Region, or BRAVE). Referrals for additional individuals who met the inclusion criteria were obtained from interviewees. Inclusion criteria required participants to be residents of Canada at the time of consent, be 18 years of age or older, be able to communicate effectively in English and provide informed verbal consent. All participants were interviewed until data saturation was reached across all participants and lack of new themes emerged based on the consensus of the two evaluators (SJ, LA), principle investigator (MG), and a consulting project manager (KM). Every third interview transcript was reviewed between evaluators to ensure thematic saturation and to triangulate results between reviewers. A $50 Visa gift card was provided to the interviewees for their participation. The consolidated criteria for reporting qualitative research (COREQ) were used to guide the reporting of the results. The study was conducted as part of a quality improvement project and received ethical approval from the University of Calgary Conjoint Health Research Ethics Board (REB21-1655).

We consulted VOMS administrators and PWUD to prepare the recruitment package, which included a verbal consent form, contact information of the study personnel, a telephone recruitment script, and a letter detailing information regarding the research study. A detailed interview script, which included questions related to naloxone kit inserts, is included in Additional file 1. The interviews were conducted over the telephone between February and March 2022. The aims and methods of the research project were explained to the research participants, and informed consent was obtained verbally. To ensure that the interview process was sound, the data collection package was used to conduct a pilot test. Telephone interviews ranged from 20 to 60 min in length and were conducted by two female evaluators (SJ, LA). The app Tapeacall was used to record the interview, and a third-party transcription service was utilized to transcribe the interviews.

Qualitative information was encoded via thematic analysis to identify themes which could help organize the perceptions and opinions shared by study participants [20]. Inductive identification of themes and subthemes was performed using thematic analysis. Responses were coded using Dedoose qualitative software by two evaluators with masters level training (SJ, LA). On the first three transcripts, coding was compared by the two evaluators to refine a codebook and ensure consistency, after which transcripts were coded independently, utilizing the refined codebook. Questions were framed in the context of Proctor’s framework, an implementation science framework for evaluating health programs [21]. Relevant codes for each evaluation question were identified, and then, coded abstracts were reviewed and analyzed for common themes.

## Results

### Sociodemographic data

Overall, our survey collected perspectives from diverse stakeholder groups, and demographic information of our key participant groups (PWUD) is summarized in Table 1. Demographic data for other participant groups were not collected but consisted of primarily urban residing respondents (*n* = 45 (87%)).

**Table 1:** Demographic information of key participant groups (PWUD)

Participant characteristics	*n* (%)
Age	
Average	38.52
Range	21–66
Gender	
Male	14(56%)
Female	10(40%)
Non-binary	1(4%)
BIPOC*	6(24%)
Indigenous	5(20%)
Urban residence	45(87%)

*Black, indigenous, and other people of color

From the interviews conducted with these participants, 4 key themes emerged, including A. acceptability of inserts in naloxone kits, B. best practices in their implementation, C. key messaging about the service, and D. facilitators to naloxone kit distribution.

### Theme A: acceptability of naloxone kit advertising

Including information about VOMS in naloxone kits is a reasonable way to promote awareness of VOMS. This included both PWUD as well as those involved in public health initiatives and care providers.

*“I think it’s probably one of the best ways to sort of penetrate into the market of substance users. The media messaging around naloxone kits is very good, and we do work with a large uptake of individuals using naloxone kits, and people who are supporters of people using substances having naloxone kits as well.” (VOMS Administrator)*

### Theme B: best practices for implementation

Information about VOMS should be provided through a dual process of having information outside of the naloxone kits but also having a business card insert within the kits as well.

When discussing the effectiveness of inserts, most interviewees suggested that information be accessible on the outside of the kits to improve awareness and reduce the risk of the information going unnoticed if kits go unopened until they are needed.

*“I think it would be most effective if it was either like a number printed on the exterior of the case or like an adhesive applied to the outside. Sometimes the kits don’t really [get] looked at until they’re needed so I think something a little more eye-catching would probably be useful.”(Community Harm Reduction Worker)*

*“So if it would be more out there like maybe on the back of a naloxone kit, yeah or something when they’re carrying it around they’re more accessible to seeing it and be like, “hey, I should spread the word about this” (PWUD without VOMS experience)*

While some respondents highlighted that information should be promoted outside the kits, having a business card insert and a QR code sticker was the preferred way to promote VOMS.

“*I think I’d be more likely to read a pamphlet or like just a little card. What might be a good idea would be saying that – you know, just very, very general quick information, like what is does, how to access it, and then having a QR link for more information if someone wants it. But the thing with only including a QR code is that there’s no guarantee that someone is going to scan.” (PWUD with VOMS experience)*

Additionally, while not reaching thematic saturation, some PWUD note that a QR code may not be appropriate for the community.

*“Yeah, a QR code is too complicated, it’s just expecting me to do too much. Especially, if I was getting high, would I really want to fuck around with something extra.” (PWUD with VOMS experience)*

### Theme C: key messaging around VOMS

When evaluating what information around VOMS should be provided, interviewees indicated that messaging should be brief and developed with people who use substances. Preferentially, it was suggested that the messaging should be no more than what can fit on a standard business card and that information should be succinct and not overwhelming.

*“You don’t want to have too much [info], because then people are going to be overloaded and they're not going to want to read it.”(PWUD Without VOMS Experience)*

When asked about types of messaging that should be included, interviewees suggested that messaging should include the name of the VOMS and phone number, a basic description regarding the service, and assurances on the confidentiality, anonymity and non-judgemental nature of the service. Lastly, messaging that PWUD don’t have to use alone was deemed necessary and useful.

*“You don’t want to overwhelm people, so I would say probably a brief script that would include the different services that they could access, like NORS (the National Overdose Response Service), like the Brave app, what their main purpose is. And you know that they're free and that they can be used from anywhere across Canada” (VOMS Administration)*

It was also important for key informants to highlight that the services are confidential and anonymous since privacy may act as a barrier for individuals using the service.

*“I would have something in there about [how] you can remain anonymous, or you know, you don’t have to submit your personal health information or anything like that to use the service, I think would be helpful. Because I think a lot of people shy away from accessing it, because they’re afraid—privacy concerns or stigma concerns.” (Community-based Harm Reduction Worker)*

Interviewees perceived the interventions to be lifesaving and suggested that they should be promoted as such. A description of why the service was important and how it potentially could prevent a fatal overdose was considered paramount.

*“People are, in my opinion, more inclined to do something if they understand a reason behind it instead of just putting something there that says “Call this when you use drugs.” Like having an explanation of like, “Listen, this is how most people die. This is what this is and this can help mitigate that.” So just the “why” as well.” (Community-based Harm Reduction Worker)*

Due to concerns about stigma as a barrier to the use of VOMS, interviewees highlighted the need to ensure that the service was non-judgemental and supportive of the client's needs. Some suggested that highlighting the service as “peer-led” (i.e., operated by people with lived experience) was equally important to help reduce stigma. However, this would not necessarily apply to all VOMS since they are not inherently peer-led.

*“So just kind of like some way to get across their vibe that they’re not going to judge you, they’re not going to make you feel like shit for calling and try to talk you out of using drugs when you’re using them but they just want to be around you. Well, like virtually, they just want to be there”. (Community-based Harm Reduction Worker)*

A brief overview of how the service works and the steps involved were considered vital information to include.

*“I would want to know first and foremost what is a virtual supervised consumption service. I would want to know what the process is to get in touch with them, whether it’s through an app or a phone line and what the steps are.” (VOMS Administration)*

Some respondents suggested that PWUD would be more likely to notice and use the service if they were informed that the service was being offered as a choice.

*“I think the more that you offer it as a choice I think that more people would use it instead of .. begging for it…. I mean yes please use it; it saves lives but like the choice is yours really like a lot of things, right. If you allow people to believe that it’s their idea they’re more likely to participate or to stand with it than if it’s kind of forced on them…” (PWUD with VOMS experience)*

Lastly, if the particular VOMS was unavailable in certain jurisdictions, it was necessary to relay that information in the pamphlet.

*“My only kind of caveat would be that not all of them are available in all service areas. So for example, if DORS were to put their contact information into naloxone kits, that would be really frustrating for people that outside of that service area” (Health Professional)*

Respondents also believed that messaging should highlight that VOMS are adjuncts to, and not replacements for, other harm reduction measures Messaging should prioritize the use of supervised consumption services for its evidence-based benefits but suggest VOMS if this access is not possible.

*“I guess the more options the better, but I don’t know that it’s a replacement for, like the SCS (supervised consumption services) that have closed down. But if it was kind of like an added option or avenue for patients who might be open to it then I think it’s good.” (Health Professional)*

### Theme D: facilitators to naloxone kit messaging

Another theme that emerged revolved around how manufacturers and distributors of naloxone kits could help inform and educate around VOMS. This included health authorities, community organizations and pharmacies, all of whom could facilitate the addition of promotional inserts on/in naloxone kits in the respondents’ opinion. One participant from the front-line harm reduction cohort highlighted:

“*Like I’m not sure how it is across the rest of Canada, but that’s – like we physically have to make our kit. So just making sure that that information is available.” (Community-Based Harm Reduction staff)*

Others highlight the importance of having PWUD involved in the design of messaging and promotional materials.

“*I think having maybe a focus group with people who use drugs about what they would want to see on the inserts, what matters to them, you know, could be helpful.” (Community-Based Harm Reduction Staff)*

Front-line staff who distribute naloxone kits were specified as key individuals who should mention VOMS and the inclusion of the inserts and QR codes at the time of distribution. Participants thought this might improve the awareness of front-line staff, PWUD and their support networks.

“*Because in order to get a naloxone kit you do have to go to the pharmacy or something like that or to the supervised consumption site. But I notice that pharmacies don’t really have that information displayed or available anywhere. So maybe giving that information to pharmacists and making sure that they have it displayed somewhere or making sure that when they’re giving someone a naloxone kit they make sure to show them that card and give them a brief summary. That would probably really, really help.” (PWUD with VOMS experience)*

Information about VOMS should be shared by other means as well through naloxone kits. Other suggested options for disseminating information about VOMS included lighters, hard cards to cut drugs, and other harm-reduction supplies. Some informants described how they had seen the name and phone number of a VOMS (those for the National Overdose Response Service specifically) on lighters and suggested that including VOMS information through harm reduction supplies and safer consumption kits might help with awareness of VOMS among the target population.

*“I think it also should be put inside needle kits and disposable pipes and other kits.” (PWUD with VOMS experience)*

## Discussion

The widespread use of naloxone kits to reverse instances of drug overdose and recent calls for scaling these programs in low- and middle-income countries alongside rural communities [18, 22] may provide an opportunity to promote harm reduction services beyond naloxone. This study explored the perspectives of diverse stakeholders on the acceptability and best practices for the implementing naloxone-kit-facilitated VOMS promotion.

Through our analysis of semi-structured interviews, we found that participants held favorable attitudes toward the use of messaging for naloxone kits. Participants highlighted a few key considerations when applying this methodology, namely including concise, non-judgemental information but disagreed on whether to include messaging inside or outside of the kits and the inclusion of the QR code. While some participants highlighted that an informational pamphlet could better describe the aspects of a virtual overdose monitoring service, others suggested that people would only open their kits in an emergency and discard any information contained within the kits. In line with this theme, previous studies have indicated that many individuals most in need of naloxone kits may already be in possession of them, and subsequent distributions are to replace used, expired, or lost kits [22]. Moreover, participants' perspectives were mixed on using a QR code as many individuals felt this method would allow for easier access to information. In contrast, others thought this method would be too complicated or did not have the appropriate technology to be able to access a QR code. Information promoted inside and outside the kits with a QR code would likely be the best strategy; however, the most important information should remain on a sticker outside the kit.

While naloxone kits are able to reach a broader audience than other harm reduction strategies including supervised consumption services and syringe programs, distribution trends toward urban individuals enrolled in opioid agonist therapy programs [24]. Indeed, strengthening awareness of VOMS would directly contribute to service efficacy regardless of the type of service offered. As recent evidence from Canada highlights, the drug overdose epidemic disproportionately impacts individuals in rural locales with up to 30% increased mortality compared to urban populations; this is partially attributed to the lack of harm reduction services in those areas [25]. While VOMS aim to provide options to those who do not have access to current harm reduction services, naloxone programs may not adequately reach communities of vulnerable rural users. Though traditionally distributed by pharmacies, harm reduction agencies and hospitals in Canada [23], other programs like Philadelphia's Mail order naloxone program may act to spread awareness of VOMS, the harms of using alone and access to naloxone kits for those most in need [26]. Respondents in the study highlighted how it was equally crucial for distributors of naloxone kits to discuss VOMS’ and indicate to individuals that they could find more information on these services inside (or outside) the kits themselves.

Participants highlighted the importance of messaging that is clear, concise, and accurate. Previous commentaries suggest that public health agencies and community stakeholders should tailor messaging to those most impacted by the drug overdose crisis, namely men who use drugs alone [27]. Furthermore, preliminary data around VOMS use including qualitative interviews and early statistics indicate that these interventions can potentially address the lack of access or comfort in using in-person harm reduction services [28, 29]. Participants highlighted the importance of advertising this service as an adjunct to supervised consumption services rather than a replacement. While VOMS help to prevent fatal overdose, they may be limited in their benefits compared to physical sites, which can provide safer consumption supplies and more rapid access to additional healthcare services [30].

Similarly, participants highlight the importance of communicating the potential improvement in client privacy provided by the service and the harms of using alone. Factors including stigma, financial motives and lack of trusted peers with whom to use their substances, the lack of knowledge about the Good Samaritan Law have been linked to engaging in these types of risky behaviors contributing to barriers to harm reduction access and may result in an individual’s decision to use alone [31]. VOMS have been suggested as an adjunct solution to supervised consumption services, and using trusted peers is another option to prevent individuals from using alone [32]. As such, increasing the use of VOMS alongside messaging about the harms of using alone may decrease fatal drug overdose for PWUD.

Lastly, interviewees highlight the value of collaboration with diverse stakeholders in the codesign and dissemination of VOMS through naloxone kits. Meaningful engagement with persons with lived and living experiences of substance use is cited as the best practice for the development of programs serving this population [33]. Furthermore, distributors including the engagement of naloxone distributors and pharmacists can promote the dissemination of VOMS information within the community. Engaging these stakeholders can help to combat stigma on behalf of communities [22] and thus may decrease barriers to accessing harm reduction.

## Limitations

There are a few limitations which must be considered when interpreting the results of our study. First, the convenience/snowball nature of the sample may have reduced the diversity of opinions in the sample, though attempts were made to recruit participants who were geographically and demographically diverse. Second, the sample may not reflect the opinions of some individuals targeted by VOMS (for example, employed people who use substances at home alone). Third, participants were recruited from individuals largely familiar with the National Overdose Response Service and thus may not reflect the opinions of those familiar with other VOMS. Fourth, the results do not prove the effectiveness of distributing information about VOMS to PWUD but only demonstrate stakeholder perceptions of appropriateness and certain best distribution practices. Furthermore, due to the composition of the study sample, responses may be biased in favor of VOMS in general. Fifth, the results do not assess any potential harm of including VOMS information in naloxone kits (for example, potential decreased visitation to supervised consumption facilities).

## Conclusions

In conclusion, our findings highlight one potential avenue for the distributing of information about VOMS through the inclusion of information on the exterior of or inside naloxone kits. This was deemed to be an acceptable approach by key stakeholders in this qualitative study. A number of suggestions for best practices using this method of information distribution were provided. Using stickers on the outside of naloxone kits and concise inserts with additional information may be a way to reach a large population of PWUD who do not engage with harm reduction services outside of naloxone kits.

## Supplementary Information

**Additional file 1.** Interview script and guide.

## Data Availability

The data that support the findings of this study are available on request from the corresponding author, M.G. The data are not publicly available due to the sensitivity of substance use and interview transcripts containing information that could compromise the privacy of research participants.

## Abbreviations

VOMS: Virtual overdose monitoring services
PWUD: People who use drugs
QR-code: Quick response-code
