# Supplementary material for: Feasibility and acceptability of inserts promoting virtual overdose monitoring services (VOMS) in naloxone kits: a qualitative study
Source: Harm Reduct J. 2023 May 8;20:64. doi: 10.1186/s12954-023-00792-z (PMC10165834; doi:10.1186/s12954-023-00792-z)
Supplement: Supplementary file 1 — Additional file 1. Interview script and guide. [file 12954_2023_792_MOESM1_ESM.docx]

Supplement 1: Interview Guide

INTERVIEW SCRIPT AND GUIDE

Only questions aiming to gather feedback about the feasibility of inserts promoting VOMS in naloxone kits have been included below.

Introduction

Thank you for agreeing to talk with me today. We would like to talk to you about something called virtual supervised consumption services. These services can help keep people safe when they use drugs and can be especially helpful for people who use alone. When individuals use some drugs, especially opioids, there can be a risk of fatal overdose. Virtual supervised consumption services can be a telephone service or a smartphone app. Each of these methods monitor people who are using substances and activate emergency services or an emergency response plan if the person using becomes unresponsive.

We would like to hear from key informants whose work or lived experience relates to the people these services are trying to help, to learn if they can be used to help other people who choose to use substances alone. Some things we would like to talk to you about are what you like and don’t like about the services as well as what you think the impacts of the services are and what should be changed to help people who use or want to use alone.

There are no right or wrong answers to my questions. We are hoping the conversation won’t take more than 45 minutes.

Please know that I do not have a personal interest in virtual supervised consumption services in general, so please feel free to speak openly and honestly. Everything you say is voluntary and will be kept confidential to the extent explained earlier.

Do you have any questions? Is it okay to proceed?

<If yes, proceed to age screening question>

<If no, ask if and what information needs to be clarified. Clarify and again ask if it is okay to proceed>

Age Verification

Before we begin, are you currently 18 years of age or older?

<If yes, proceed to first question>

<If no, thank the person for their time and let them know that the person must be 18 years of age to participate>

| INTERVIEW GUIDE | |
| --- | --- |
| Question | Probe |
| Do you recall ever seeing information of virtual supervised consumption services in naloxone kits? | If yes, do you recall what that naloxone sticker/insert said about virtual supervised consumption services?  Do you think the naloxone kit inserts included all of the relevant and necessary information needed, or was there anything missing?  Do you think putting information about VSC services in naloxone kits is a good way to spread awareness about the services? |
| Do you have any suggestions for information that should be included in naloxone kits to improve awareness of virtual supervised consumption services? | Any specific messaging? |
| Do you think there is high awareness of VOMS among those who choose to use substances? | If yes, why? |
| Any other ideas on how to promote awareness of virtual supervised consumption services? | What are the barriers to promoting awareness of VOMS? |
| Demographic Questions | |
| What organization do you work for? |  |
| What type of organization do you work for? |  |
| What is your role at the organization? |  |
| In which province or territory are you currently residing? |  |
| Do you consider the place you currently live to be urban or rural? |  |

Thank you so much for your time. Please feel free to contact me if you think of anything else that you’d wish to include. Take care
